# Health-related quality of life in long-term head and neck cancer survivors: a comparison with general population norms

**DOI:** 10.1054/bjoc.2000.1576

**Published:** 2001-01

**Authors:** E Hammerlid, C Taft

**Affiliations:** Department of Otolaryngology and Head and Neck Surgery, Sahlgrenska University Hospital, Göteborg University, Göteborg, Sweden; Health Care Research Unit, Department of Internal Medicine, Sahlgrenska University Hospital, Göteborg University, Göteborg, Sweden

**Keywords:** head and neck cancer, survivors, health-related quality of life (HRQL), SF-36, EORTC QLQ-H&N35

## Abstract

To examine the health-related quality of life (HRQL) in long-term head and neck (H&N) cancer survivors compared with general population norms. HRQL was assessed with three standardized questionnaires: the SF-36 Health Survey (Short Form 36) and the EORTC QLQ-C30 and QLQ-H&N35 (European Organization for Research and Treatment of Cancer Quality of Life Questionnaire, -Core 30 and -Head and Neck 35 cancer module). Altogether 135 H&N cancer patients (mean age 62 years, 31% females) of 151 survivors (89% acceptance) from a longitudinal HRQL study (*n* = 232) were included 3 years after diagnosis. The H&N cancer patients' SF-36 scores did not differ significantly from those of an age- and sex-matched sample (*n* = 871) from the Swedish normative population, except on the role-physical functioning scale. On the other hand, treatment-related side-effects and disease-specific problems (e.g., swallowing, local pain and dry mouth) measured by the H&N cancer module were, with few exceptions, significantly worse than norm values. Gender comparisons revealed that female H&N cancer patients generally scored better than the norms on both the SF-36 and the EORTC QLQ-C30, while the male patients scored significantly worse on most SF-36 scales. Patients ≥65 years more often scored worse than the norm than did patients <65. Clinically relevant differences were found on the majority of SF-36 scales in comparison of tumour sites, however, comparisons of patients with small (stage I+II) versus advanced (stage III+IV) tumours revealed few differences. Three years after diagnosis H&N cancer patients still suffer significant functional limitations/problems related to their disease and its treatment but these problems do not generally affect their overall HRQL. Tumour stage no longer differentiates HRQL at 3 years, however, factors related to the patients' age, gender and location of the tumour appear to have bearing on their reported health status. © 2001 Cancer Research Campaign http://www.bjcancer.com


*British Journal of Cancer* (2001) **84**(2), 149–156
© 2001 Cancer Research Campaign
doi: 10.1054/ bjoc.2000.1576, available online at http://www.idealibrary.com on IDEAL®

### Health-related quality of life in long-term head and neck cancer survivors: a comparison with general population norms

**E Hammerlid[1] and C Taft[2]**

[1]Department of Otolaryngology and Head and Neck Surgery, Sahlgrenska University Hospital, Göteborg University, Göteborg, Sweden;
[2]Health Care Research Unit, Department of Internal Medicine, Sahlgrenska University Hospital, Göteborg University, Göteborg, Sweden

**Summary** To examine the health-related quality of life (HRQL) in long-term head and neck (H&N) cancer survivors compared with general population norms. HRQL was assessed with three standardized questionnaires: the SF-36 Health Survey (Short Form 36) and the EORTC QLQ-C30 and QLQ-H&N35 (European Organization for Research and Treatment of Cancer Quality of Life Questionnaire, -Core 30 and -Head and Neck 35 cancer module). Altogether 135 H&N cancer patients (mean age 62 years, 31% females) of 151 survivors (89% acceptance) from a longitudinal HRQL study ($n$ = 232) were included 3 years after diagnosis. The H&N cancer patients' SF-36 scores did not differ significantly from those of an age- and sex-matched sample ($n$ = 871) from the Swedish normative population, except on the role-physical functioning scale. On the other hand, treatment-related side-effects and disease-specific problems (e.g., swallowing, local pain and dry mouth) measured by the H&N cancer module were, with few exceptions, significantly worse than norm values. Gender comparisons revealed that female H&N cancer patients generally scored better than the norms on both the SF-36 and the EORTC QLQ-C30, while the male patients scored significantly worse on most SF-36 scales. Patients ≥65 years more often scored worse than the norm than did patients <65. Clinically relevant differences were found on the majority of SF-36 scales in comparison of tumour sites, however, comparisons of patients with small (stage I+II) versus advanced (stage III+IV) tumours revealed few differences. Three years after diagnosis H&N cancer patients still suffer significant functional limitations/problems related to their disease and its treatment but these problems do not generally affect their overall HRQL. Tumour stage no longer differentiates HRQL at 3 years, however, factors related to the patients' age, gender and location of the tumour appear to have bearing on their reported health status. © 2001 Cancer Research Campaign http://www.bjcancer.com

**Keywords:** head and neck cancer; survivors; health-related quality of life (HRQL); SF-36; EORTC QLQ-H&N35

Approximately 2–3% of all newly diagnosed cancer in Sweden is H&N cancer and its incidence is estimated to increase by approximately 2% annually (Einhorn et al, 1996). Although tumour sites (larynx, oral cavity, oropharynx, sinus and nose and the salivary glands) are known to affect crucial functions, such as the ability to breath, speak, eat and smell, until recently little has been known about how such impairments impact on the HRQL of H&N cancer patients.

The first HRQL studies of H&N cancer patients were cross-sectional or retrospective (Pruyn et al, 1986; de Boer et al, 1999), but during the last few years prospective studies have also been published (Jones et al, 1992; List et al, 1996; Deleyiannis et al, 1997; Funk et al, 1997; Hammerlid et al, 1997; Hammerlid et al. 1997; Morton, 1997; Hammerlid et al, 1998; Hammerlid et al, 1998; Bjordal et al, 1999; de Graeff et al, 1999; de Graeff et al, 1999; Deleyiannis et al, 1999; List et al, 1999; Rogers et al, 1999). These studies have been performed with validated questionnaires and have consistently shown that H&N cancer patients' HRQL is significantly below norm values at diagnosis and decreases during and immediately after treatment. During this period, the patients not only have major problems with pain and nutrition but are also limited in daily physical and social functioning. However, within the first year after diagnosis most of these mentioned problems/functions return to their pretreatment values, except symptoms and problems specifically related to treatment, such as xerostomia and reduced taste and smell. On the other hand, mood disorder, especially anxiety, have been found to be less common one year after diagnosis (Hammerlid et al, 1999).

In a study published by Funk (Funk et al, 1997), the HRQL of a H&N cancer sample was compared with age-matched, US population norms for the SF-36 (Ware et al, 1993). In this study, it was shown that the H&N cancer patients (particularly the younger ones) scored significantly lower for most on the HRQL domains measured, both at diagnosis and after 6 months.

Although the short-term and intermediate effects of H&N cancer and its treatment are well documented, we know very little about the HRQL of H&N cancer survivors more than 12 months after diagnosis. An important question is to what degree they recover normal health status after rehabilitation, i.e. how long-term H&N cancer survivors' health status compares with that of their contemporaries in the general population. A study was therefore performed to examine the HRQL of a large group of H&N cancer survivors 3 years after diagnosis compared with population norms. For this purpose, the generic IQOLA SF-36 Health Survey was chosen with its

*Received 27 April 2000*
*Revised 31 August 2000*
*Accepted 11 October 2000*

*Correspondence to:* E Hammerlid normative database for the Swedish population (Sullivan et al, 1994). The secondary aim was to assess the specific tumour burden and treatment side-effects. For this purpose, the EORTC QLQ-C30 (Aaronson et al, 1993) and QLQ-H&N35 were used (Bjordal et al, 1999).

### MATERIAL AND METHOD

### Study design

Adult patients with diagnosed and untreated primary H&N cancer (ICD-9 141–148, 160, 161 and 196) were invited consecutively to participate in a longitudinal quality of life study at Sahlgrenska University Hospital, Göteborg, during 1993–95. Survivors were phoned 3 years after diagnosis and asked to complete the battery of quality of life questionnaires once more. Patients disrupting the previous study were also asked to participate. Those who were unable to answer the HRQL questionnaires due to senile dementia, mental disturbance or severe intercurrent disease were excluded. No other exclusion criteria were used.

Patients who agreed to participate were mailed the questionnaires. They were reminded once if they did not return the questionnaire within 10 days.

Tumour location according to the ICD-9, TNM classification (UICC-1987), treatment and clinical data including comorbidity and Karnofsky Performance Status (Karnofsky et al, 1948) were noted, together with the patient's present tumour status. The study was approved by the local ethics committee.

### QL questionnaires

#### *SF-36 health survey*

The SF-36 is a generic short-form measure of functional health and well-being. It has been extensively applied in comparing general and specific populations, estimating the burden of disease and measuring the effectiveness of treatments (Ware and Sherbourne 1992; Ware et al, 1993). The Swedish version was first made available in 1992 and Swedish population norms were published in 1994 (Sullivan et al, 1994, 1995). The questionnaire consists of 8 health domains: physical health (10 questions), role limitations due to physical problems (4 questions), bodily pain (2 questions), general health (5 questions), vitality (4 questions), social functioning (2 questions), role limitations due to emotional problems (3 questions), mental health (5 questions) and a question about perceived change of health during the last year. A score between 0 (worst) and 100 (best) is calculated for each domain using a standardized scoring system (Ware et al, 1993).

#### *EORTC QLQ-C30*

This questionnaire (version 1.0) is a tumour-specific, patient-based instrument designed for self-administration. The cross-cultural validity and the psychometric properties are considered satisfactory (Aaronson et al, 1993; Osoba et al, 1994; Hjermstad et al, 1995). It has previously been used in HRQL studies of H&N cancer (Bjordal and Kaasa 1992; Jones et al, 1992; Bjordal et al, 1994; Bjordal and Kaasa 1995; Bjordal et al, 1995; Hammerlid et al, 1997a, 1997b; Hammerlid et al, 1998,a,b,c). The questionnaire comprises 5 functional scales: physical functioning (5 questions), role functioning (2 questions), emotional functioning (4 questions), cognitive functioning (2 questions) and social functioning (2 questions). There are three symptom scales: fatigue (3 questions), nausea and vomiting (2 questions) and pain (2 questions) and 6 single items relating to dyspnoea, insomnia, loss of appetite, constipation, diarrhoea and financial difficulties. It also includes a global health status/QL scale (2 questions). A one-week time frame is employed. The HRQL scores are calculated according to the EORTC QLQ-C30 scoring manual (Fayers et al, 1995). All scales and single-item scores are transformed into a score from 0 to 100. A high score for a functional scale and for the global health status/QL scale represents a high level of functioning, while a high score for a symptom scale or single item represents a high level of symptoms.

#### *EORTC QLQ-H&N35*

To measure symptoms and problems related to tumour location and treatment, the EORTC head and neck cancer module, QLQ-H&N35, was used (Bjordal et al, 1994, 1999). The instrument comprises seven subscales: pain (4 questions), swallowing (5 questions), senses (2 questions), speech (3 questions), social eating (4 questions), social contact (5 questions) and sexuality (2 questions). There are 10 single items relating to problems with teeth, dry mouth, cough, opening the mouth wide, sticky saliva, weight loss, weight gain, use of nutritional supplements, feeding tubes and painkillers.

In the present study an interim version of the module was used because the final version was received after the start of this study. The interim version differed from the final version in not including a question on weight gain and the question about sticky saliva was worded slightly different. The module is scored according to the same scoring system as the EORTC QLQ-C30.

#### *Study-specific questionnaire*

This questionnaire contained 8 self-reported questions relating to family situation, education, work and smoking habits.

### Reference samples

Three different general population samples were used.

(i) *SF-36 reference group*. An age and gender-matched sample comprising 871 individuals was randomly selected from the Swedish SF-36 normative database consisting of 8930 subjects (Sullivan et al, 1994).

(ii) *EORTC QLQ-C30 reference group*. Age and gender-adjusted reference values ($n$ = 276) for a Norwegian population sample were obtained from published norm tables (Hjermstad et al, 1998a, b). The tables give mean scores for the total group, by gender and by age group (10 year intervals), but do not provide measures of variance.

(iii) *EORTC QLQ-H&N35 reference group*. An age and gender-adjusted reference group ($n$ = 270) from a population sample from our own department was used. This population sample consisted of 700 randomly selected inhabitants of western Sweden. They answered the first 22 questions of the EORTC QLQ-H&N 35 as part of an investigation on the prevalence of dysphagia in the population (Hammerlid, 1997).

### Patients

A total of 232 patients were included in the first part of the HRQL-study 1993–95, 151 (65%) were alive 3 years after diagnosis. All 151 patients were found and contacted. Of these, 135 (89%) met the inclusion criteria and agreed to take part in the study. The mean

© 2001 Cancer Research Campaign

**Table 1** Patient characteristics

| | All | Sex | | Stage* | | Age | |
|---|---|---|---|---|---|---|---|
| | | **Female** | **Male** | **I + II** | **III + IV** | **<65** | **>65** |
| Number (%) | 135 | 42 (31) | 93 (69) | 66 (55) | 55 (45) | 73 (57) | 56 (43) |
| Mean age | 62 | 60 | 63 | 60 | 63 | 52 | 75 |
| *Stage** | | | | | | | |
| Stage I + II | 66 (55) | 23 (59) | 43 (52) | – | – | 38 (57) | 25 (52) |
| Stage III + IV | 55 (45) | 16 (41) | 39 (48) | – | – | 29 (43) | 23 (48) |
| *Tumor site* | | | | | | | |
| Larynx | 28 (21) | 5 (12) | 23 (25) | 21 (30) | 7 (13) | 12 (16) | 14 (25) |
| Oral cavity | 40 (30) | 16 (38) | 24 (26) | 27 (41) | 11 (20) | 23 (32) | 16 (29) |
| Pharyngeal | 35 (26) | 11 (26) | 24 (26) | 9 (14) | 26 (47) | 23 (32) | 9 (16) |
| Other tumour sites | 32 (23) | 10 (24) | 22 (23) | 9(14) | 11 (20) | 15 (20) | 17 (30) |
| *Treatment*** | | | | | | | |
| Surgery | 11 (8) | 6 (14) | 5 (5) | 7 (11) | 1 (2) | 6 (8) | 5 (9) |
| Radiation | 39 (29) | 7 (17) | 32 (34) | 32 (48) | 7 (13) | 18 (25) | 18 (32) |
| Rad+Surg | 39 (29) | 18 (43) | 21 (23) | 23 (35) | 8 (14) | 22 (30) | 16 (29) |
| Chemo+Rad+Surg | 11 (8) | 3 (7) | 8 (9) | 1 (1) | 7 (13) | 6 (8) | 4 (7) |
| Chemo+Rad | 35 (26) | 8 (19) | 27 (29) | 3 (5) | 32 (58) | 21 (29) | 13 (23) |

Note: The number together with per cent in parentheses is given. Rad: Radiation therapy, Surg: Surgery, Chemo: Chemotherapy.*: Stage is missing for 14 patients, ** Treatment, age < or > 65 years is missing for 6 patients.

age of the 135 H&N cancer patients was 62 years (18–83) and 42 patients were female (31%). Less than half of the patients were retired (*n* = 59, 44%) and 76 of the patients were less than 65 years old (56%) at the time for diagnosis.

The most common tumour site was the oral cavity (40 patients), followed by the pharyngeal (35 patients), ‘other’ tumour sites (salivary glands, unknown primary, sinus and nose carcinoma, 32 patients) and the larynx (28 patients).

At the time of diagnosis 30% of the patients had a stage I disease, 24% had stage II, 21% had stage III and 25% had stage IV (stage was missing for 14 patients, the majority of whom had sinus or nose carcinoma). Almost all patients (91%) had had radiation therapy as part of their treatment and 37% of the patients had been treated with interstitial radiation therapy. Chemotherapy had been given to 34% of the patients (cis-platinol in combination with 5-flourouracil). The different combinations of treatment for the entire study group and subgroups of patients are shown in Table 1.

At the 3-year follow-up 126 of the patients had no signs of relapse, 5 patients had been treated for a relapse but were tumour free at the assessment point and 4 patients had an active disease (one patient with oral cancer and 3 patients with ‘other tumours’).

At the time of diagnosis 33% of the patients lived alone, 17% had children living in their household, 39% were working, 51% were retired and the rest were either unemployed, students or homemakers (10%).

8% of the patients had had a previous malignancy, 13% were under treatment for heart disease, 7% for pulmonary disease and 18% for another disease.

### Statistical methods

For descriptive purposes, we used means and 95% confidence intervals for the mean. For comparisons between groups, Fisher’s non-parametric permutation test was applied (Bradley, 1968). Fisher’s exact test was used for comparison between proportions and Pitman’s non-parametric permutation test for correlation analysis (Bradley, 1968). The significance level was set at 5% throughout. For each SF-36 scales a stepwise regression analysis was performed with the SF-36 scales as dependent variables and sex, age, disease, stage, tumour site and treatment as independent.

### Strategy of analysis

To aid in the interpretation of the results, clinical significance of between-group differences is reported together with statistical significance. Statistical and clinical significance are two very distinct, yet equally important ways of interpreting group differences. Statistical significance refers to the probability that a difference occurred by chance alone. In contrast, clinical significance refers to the practical implications of the difference in terms of its relative impact on health or well-being. Statistical significance depends not only on the size of the difference between the groups, but also on the amount of variation within the groups and on the number of patients in the study. Thus, clinically trivial differences can be statistically significant if, for example, the sample size is sufficiently large, and conversely, clinically important differences can be statistically non-significant if the study lacks power. While standards for statistical significance have wide acceptance and application, criteria for clinical significance are less well-defined. We have applied criteria for interpreting score differences that have been proposed for the instruments used in this study. For the EORTC questionnaires, a 10 point change in score has been suggested by King and Osoba (King, 1996; Osoba et al, 1998) as clinically significant. The same criterion was used both for the EORTC QLQ-C30 and EORTC QLQ-H&N35, even though this criterion has been suggested only for the core questionnaire. For the SF-36, we used a 5 point difference as an indicator of clinically and socially relevant change, as suggested by Ware (Ware et al, 1993).

*© 2001 Cancer Research Campaign*

**Table 2** Comparison of the results from the SF-36 for the study sample and the Swedish population

| | H&N cancer patients ($n$ = 135) | Population sample ($n$ = 871) | $P$ value |
|---|---|---|---|
| Physical functioning | 75 (71–80) | 79 (77–81) | 0.110 |
| Role-physical functioning | 64 (57–71) | 74 (71–76) | 0.008 |
| Bodily pain | 73 (69–77) | 71 (69–72) | 0.346 |
| General health | 67 (63–71) | 70 (69–72) | 0.077 |
| Vitality | 67 (63–72) | 67 (66–69) | 0.985 |
| Social functioning | 84 (80–88) | 87 (86–89) | 0.140 |
| Role-emotional functioning | 77 (70–84) | 82 (80–84) | 0.119 |
| Mental health | 79 (75–82) | 81 (79–82) | 0.328 |

The table shows mean values with 95% confidence intervals. $n$ = number of patients.

### RESULTS

### Results from the SF-36

Means and confidence intervals of the 8 SF-36 scales for the study sample and reference group are shown in Table 2. The H&N cancer patients scored worse or equal (–10–0 points) to the reference group on all domains, except bodily pain. The difference was clinically relevant (≥5 points) on two of the domains: the role-physical functioning (△10 points, $P$ = 0.008) and the role-emotional functioning (△5 points, $P$ = 0.11) but only the role-physical functioning was statistically significant.

### *Female and male patients versus population norms*

For the comparison of female cancer patients versus female reference data and male patients versus male reference data two new groups of sex- and age-matched SF-36 normative data were used. Female H&N cancer patients scored the same or better than the reference group on all 8 SF-36 domains, Figure 1A. A clinically and statistically significant difference between the two groups was found for vitality (△8 points, $P$ = 0.05). The other domain with a difference of ≥5 points, bodily pain, was not statistically significant ($P$ = 0.15).

The opposite trend was found for the males, i.e. the population sample scored better than the H&N cancer patients on 7 of the 8 SF-36 scales, Figure 1B. The differences were clinically relevant for 5 and statistically significant for 4 of the scales. The largest difference was found for role-physical functioning (△15 points, $P$ < 0.001), followed by role-emotional functioning (△8 points, $P$ = 0.029), physical functioning (△7 points, $P$ = 0.011), general health (△6 points, $P$ = 0.023) and social functioning (△5 points, $P$ = 0.064).

Comparisons between male and female H&N cancer patients showed females to score better than males on all scales except mental health. Clinically important differences were found for physical functioning, role-physical functioning, general health, vitality and for role-emotional functioning but none was statistically significant.

In the stepwise regression analysis, gender was forced into the model but did not explain a significant proportion of the variance in any SF-36 scale.

### *Patients below and above retirement age*

Figures 2A and 2B illustrate comparisons of patients below and above retirement age (65 years) with SF-36 normative data for sex- and age-matched subjects.

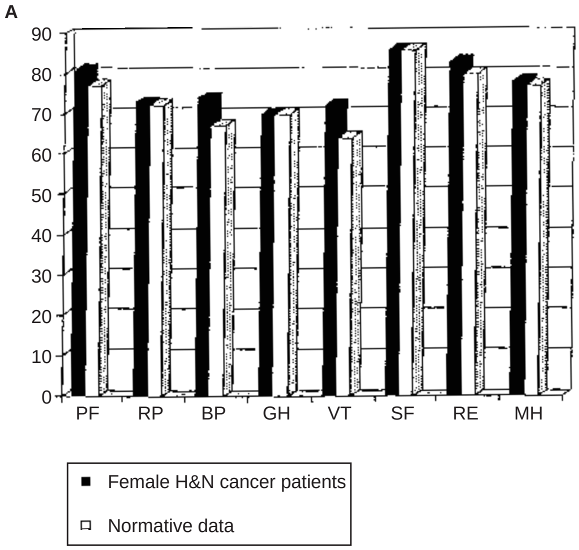

**Figure 1A** The results from the SF-36 for female H&N cancer patients ($n$ = 42) 3 years after diagnosis compared to age-matched female normative data. The higher the score, the better functioning. PF = Physical functioning, RP = Role-Physical, BP = Bodily Pain, GH = General Health, VT = vitality, SF = Social Functioning, RE = Role-Emotional, MH = Mental Health

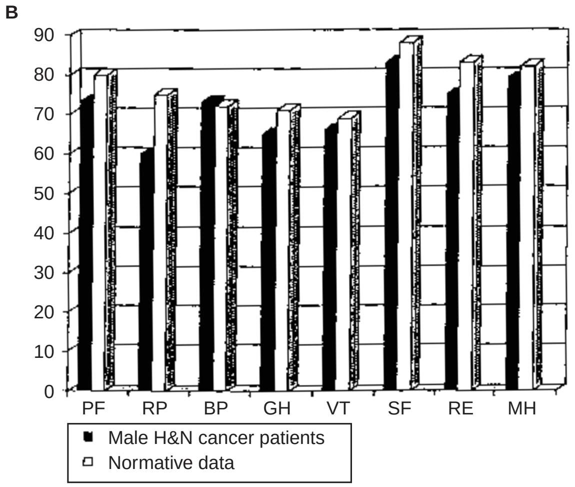

**Figure 1B** The results from the SF-36 for male H&N cancer patients ($n$ = 93) 3 years after diagnosis compared to age-matched male normative data. The higher the score, the better functioning. PF = Physical Functioning, RP = Role-Physical, BP = Bodily Pain, GH = General Health, VT = vitality, SF = Social Functioning, RE = Role-Emotional, MH = Mental Health

© 2001 Cancer Research Campaign

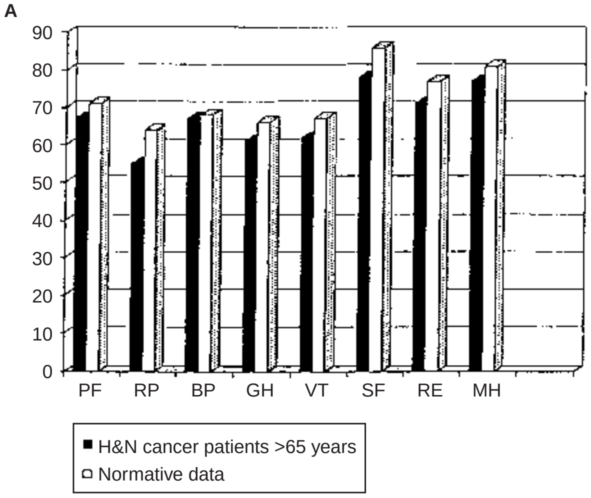

**Figure 2A** The results from the SF-36 for H&N cancer patients >65 years 3 years after diagnosis compared to age- and sex-matched normative data. The higher the score, the better functioning. PF = Physical Functioning, RP = Role-Physical, BP = Bodily Pain, GH = General Health, VT = vitality, SF = Social Functioning, RE = Role-Emotional, MH = Mental Health

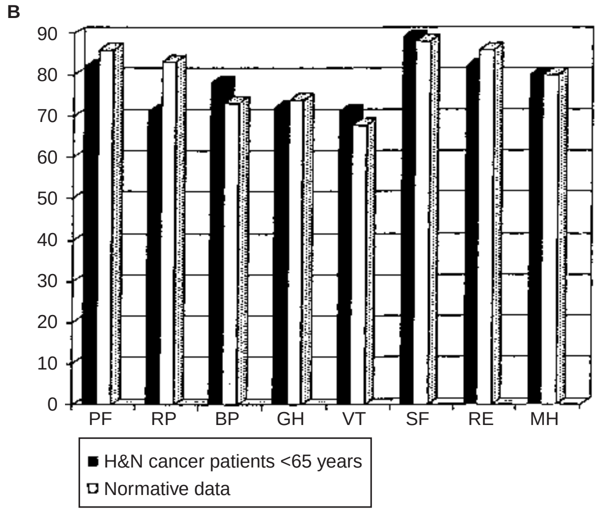

**Figure 2B** The results from the SF-36 for H&N cancer patients < 65 years 3 years after diagnosis compared to age- and sex-matched normative data. The higher the score, the better functioning. PF = Physical Functioning, RP = Role-Physical, BP = Bodily Pain, GH = General Health, VT = vitality, SF = Social Functioning, RE = Role-Emotional, MH = Mental Health

**Table 3** Results from the SF-36 for subgroups of head and neck cancer patients three years after diagnosis

| | **Tumour site** | | | | **Stage** | |
|---|---|---|---|---|---|---|
| | **Larynx (*n* = 28)** | **Oral cavity (*n* = 40)** | **Pharyngeal (*n* = 35)** | **Other (*n* = 32)** | **I+II (*n* = 66)** | **III+IV (*n* = 55)** |
| Physical functioning | 74 (63–85) | 75 (67–84) | 75 (66–84) | 77 (67–86) | 74 (67–81) | 76 (70–83) |
| Role-physical functioning | 63 (46–79) | 63 (48–77) | 71 (57–85) | 59 (43–74) | 63 (53–74) | 65 (53–77) |
| Bodily pain | 76 (66–86) | 71 (62–80) | 78 (63–81) | 74 (66–83) | 76 (69–82) | 70 (63–76) |
| General health | 66 (56–76) | 68 (60–76) | 66 (57–75) | 66 (59–74) | 68 (62–75) | 65 (59–71) |
| Vitality | 71 (60–81) | 66 (58–74) | 67 (57–76) | 67 (60–74) | 68 (62–75) | 68 (61–74) |
| Social functioning | 90 (85–96) | 81 (72–91) | 81(74–89) | 85 (78–92) | 84 (78–90) | 84 (78–90) |
| Role-emotional functioning | 83 (69–97) | 77 (63–91) | 78 (65–91) | 72 (58–87) | 76 (66–87) | 79 (70–89) |
| Mental health | 83 (75–90) | 76 (69–83) | 78 (70–85) | 80 (74–86) | 82 (76–85) | 77 (71–83 |

The table shows the mean values and 95% confidence intervals. *n* = number of patients.

**Table 4** Results from the EORTC QLQ-H&N35. A comparison of the head and neck cancer patients with a sample from the Swedish population

| | **H&N cancer patients (*n* = 135)** | **Population sample (*n* = 270)** | ***P* value** |
|---|---|---|---|
| *H&N35 scales* | | | |
| Pain | 14 (11–18) | 3 (2–4) | <0.0001 |
| Swallowing | 11 (7–14) | 3 (2–4) | <0.0001 |
| Senses | 20 (16–25) | 3 (2–5) | <0.0001 |
| Social eating | 11 (8–15) | 1 (1–2) | <0.0001 |
| *H&N35 single items* | | | |
| Problems with teeth | 21 (15–26) | 12 (9–15) | <0.01 |
| Opening mouth wide | 17 (12–22) | 1 (0–2) | <0.0001 |
| Dry mouth | 47 (41–53) | 17 (14–20) | <0.0001 |
| Mucus production | 18 (13–23) | 7 (5–9) | <0.0001 |
| Cough | 17 (13–21) | 19 (16–22) | 0.79 |
| Feeling ill | 11 (7–14) | 10 (7–12) | 0.34 |

The table shows mean values with 95% confidence intervals. The higher the score, the more problems. *n* = number of patients.

Scores for patients below retirement age were similar to reference values (± △4 points) on all scales except role-physical functioning (–△12 points, $P$ = 0.012) where the H&N cancer patients scored worse and bodily pain (+△5 points, $P$ = 0.117) where the cancer patients scored better. Comparisons between retired patients and their counterparts in the reference sample revealed clinically important differences in favour of the reference group on 5 of the 8 scales but only one was statistically significant. The largest score differences were found for role-physical functioning (–△9 points, $P$ = 0.110) and social functioning (–△8 points, $P$ = 0.020). Other clinically relevant differences (≥5 points) were found for role-emotional functioning, general health and vitality, but these were not statistically significant.

### Different tumour sites and stage

Clinically important differences between the worst and best SF-36 scales scores were found when the different tumour sites were compared (Table 3). The role-physical functioning and role-emotional functioning varied most. Laryngeal cancer patients scored highest on 4 of the scales (mental health related) while the oral cancer group scored worst on 3.

Only a few clinical relevant differences were noted between patients with small (stage I+II) and large (stage III+IV) tumours (Table 3). Patients with small tumours scored higher on the bodily pain and mental health scales (+△6 respectively +△5 points) but these differences were not statistically significant.

### Results from the EORTC QLQ-C30 and QLQ-H&N35

The results from the EORTC QLQ-H&N35 are shown in Table 4. The H&N cancer patients scored significantly worse compared to the population sample on all scales and single items examined, except for cough and feeling ill. Most of the differences were both clinically (difference ≥10 points) and statistically significant. The largest differences were found for dry mouth, senses and opening the mouth wide.

In order to corroborate the gender differences found for the SF-36, the results from the EORTC QLQ-C30 for the females and males were compared with Norwegian reference data (Table 5). These reference data were only available for certain age groups (Hjermstad et al, 1998). Age 60–69 was chosen for comparison since the mean age in the patient sample was 60 and 63 for the females and males, respectively.

The female cancer patients scored better on 13 of the 15 scales and single items in the QLQ-C30 compared to the female reference data. Five of the scores showed a clinically significant difference (⩾10 points) in favour of the cancer patients: physical functioning, fatigue, dyspnoea, insomnia and pain.

The male cancer patients scored better on 7 of the 15 scales and items compared to the reference data but none of the differences reached ≥10 points.

When the scores were compared between the female and male cancer patients, the females scored better on 11 of the 15 scales and single items but only the difference on dyspnoea was clinically important.

### DISCUSSION

Previous research has shown that the HRQL of H&N cancer patients is poor at diagnosis compared to a normative population sample (Funk et al, 1997) and deteriorates during and immediately after treatment. However, within a year after diagnosis most of the general functions and treatment-related side-effects return to their pre-treatment values (Hammerlid et al, 1997a,b, 1998, 1999; de Graeff et al, 1999a,b). Little is known about this patient group beyond one year post-diagnosis. Consequently, this study was conducted to evaluate the long-term (3 years) HRQL of H&N cancer survivors to determine if their levels of mental and physical functioning are comparable to population norms.

In general, the results of this study indicate that the general health status of long-term H&N cancer survivors is comparable to that of age-and gender-matched population norms. Only one of the 8 SF-36 health domains differed significantly from the norm and none of the domains measured by the cancer-specific EORTC QLQ-C30 showed clinically important deviations below the reference values (Tables 2 and 5).

On the other hand, gender comparisons with normative data revealed some interesting and unexpected results. The female cancer patients tended to score better than female norms, both on the SF-36 and the EORTC QLQ-C309 (Figure 1A and Table 5) while the male H&N cancer patients reported worse HRQL for the majority of scales than male norms for the SF-36 and EORTC QLQ-C30 (Figure 1B and Table 5). The differences between the male cancer patients and the male normative group were significant for most of SF-36 scales.

When the females and males were compared, the females had a tendency to score better than the males. These findings were also unexpected since females have been consistently shown to report poorer HRQL than males in both general populations and in different clinical groups (Sullivan et al, 1994; Chin and Goldman, 1998; Hjermstad et al, 1998; Osborne et al, 1998). Consequently, it was considered important to see if the patients' gender was a major determinant of reported health status, or if the effects of gender were confounded by other variables, especially since males and females differed in mean age and distribution of tumour sites and treatment modality. As a first step, baseline male and female

**Table 5** A comparison between the female and male head and neck cancer patients and Norwegian population values

| | Female | | Male | |
|---|---|---|---|---|
| | **Cancer patients** | **Population sample** | **Cancer patients** | **Population sample** |
| *n* | 42 | 142 | 93 | 134 |
| Age | mean 60 | 60–69 | mean 63 | 60–69 |
| Functional scales* | | | | |
| Physical functioning | 89 (82–96) | 78 | 85 (80–89) | 89 |
| Role functioning | 87 (78–96) | 89 | 83 (77–89) | 90 |
| Social functioning | 91 (85–97) | 83 | 86 (81–90) | 81 |
| Emotional functioning | 85 (79–91) | 83 | 86 (82–90) | 85 |
| Cognitive functioning | 91 (86–96) | 86 | 86 (83–96) | 83 |
| Global quality of life | 74 (66–81) | 69 | 73 (69–78) | 74 |
| Symptom scales/single items** | | | | |
| Fatigue | 18 (10–25) | 33 | 24 (20–29) | 24 |
| Pain | 12 (5–18) | 32 | 16 (12–21) | 21 |
| Nausea and vomiting | 2 (0–4) | 4 | 3 (1–4) | 2 |
| Dyspnoe | 10 (4–16) | 20 | 20 (15–25) | 16 |
| Insomnia | 18 (9–26) | 33 | 18 (13–23) | 19 |
| Loss of appetite | 15 (6–23) | 6 | 10 (5–15) | 4 |
| Constipation | 15 (5–23) | 16 | 9 (5–15) | 9 |
| Diarrhoea | 2 (0–4) | 9 | 5 (2–18) | 8 |
| Financial difficulties | 9 (3–15) | 12 | 12 (6–17) | 13 |

Note: *Higher score means better functioning. **Higher score means more problems.
*n* = number of patients.

 *© 2001 Cancer Research Campaign*

scores on the EORTC QLQ-C30 and QLQ-H&N35 (SF-36 was not a baseline instrument) were compared to see if they differed at outset. At diagnosis, the males scored better on 10 of the 15 EORTC QLQ-C30 scales and single items, with clinically significant differences on emotional functioning and appetite loss (data not shown). On the H&N module, the only significant difference was on sexual functioning, where the males again scored better. No differences could be found regarding the incidence of comorbid conditions. Stepwise regression analyses were then performed with each of the 8 SF-36 scales as dependent variables and sex, age, disease stage, tumour site, treatment modality and number of comorbidities as independents. Although sex was forced into each of the models, it could not account for a significant proportion of the variance in any of the SF-36 scales. In fact, none of the background variables contributed more than 4% of the variance in any scale. Thus, although the females, on average, reported better health status than their male counterparts at follow-up, other (unexplained) factors besides gender determined the SF-36 scale scores. Further research with a larger study group should be conducted to identify such factors.

Almost all functions and problems measured by the H&N cancer-specific EORTC QLQ-H&N35 were significantly worse for the H&N cancer patients than the population sample (Table 4). Thus, despite significant problems with important functions like swallowing, social eating and localized pain, the patients reported normal general functioning and mental health 3 years after diagnosis.

A possible explanation for this apparent incongruity between general health status and H&N cancer-related limitations/problems reported by H&N cancer survivors concerns the inherent differences between disease-specific versus generic health status instruments. As the name implies, disease-specific instruments, such as the EORTC QLQ-H&N35, are designed to tap symptoms, problems and limitations distinctly associated with a particular disease, while generic instruments, such as the SF-36, cover general health domains found important and applicable in general populations. The two thus contribute complementary information on different aspects of overall health status. Where the first is sensitive to specific disease or treatment burdens, the second informs about the impact of such burdens on the patient's overall physical functioning and mental well-being. In other words, although the H&N cancer patients in this study still experience considerable problems directly associated with their disease and/ or its treatment, they have successfully adjusted to living with their problems and thus assess their overall physical and mental health at levels comparable to norms.

The pain scales common to the 3 instruments may be used to illustrate the inherent differences between disease-specific versus generic health status instruments. On the EORTC QLQ-H&N35 the patients reported significantly more pain than the population, while on both the SF-36 and EORTC QLQ-C30 they scored at or better than norm levels (Tables 2, 4 and 5). The first instrument assesses the intensity of pain localized to the head and neck region, while in the EORTC QLQ-C30 and SF-36 questions are asked about both the intensity of non-specified pain and its impact on one's ability to work or perform daily activities. This may be interpreted to mean that although the patients suffer from substantial localized pain, it does not interfere with the performance of their daily activities. It is important to note, however, that the cancer patients scored worse than the norm on the SF-36 role physical functioning scale (Table 2). This implies that the patients felt limited, for reasons other than pain, by their physical health in carrying out daily activities.

In a previous study of mental health in head and neck cancer patients the prevalence of depression was reported to be 17–29% one year after diagnosis (Hammerlid et al, 1999). After an initial period of deterioration during and just after treatment, patients tended to report improved mental health at 6 months and fully return to pre-treatment levels at 12 months (Funk et al, 1997; Hammerlid et al, 1999). Our results are optimistic in that they indicate that in the long-term mental health continues to improve, reaching levels corresponding to norms for the general population.

Another finding was that disease stage at diagnosis had little impact on the HRQL after 3 years later (Table 3). This result was unexpected since in our previous studies, patients with more advanced diseases (Stage III or IV) had worse HRQL at diagnosis and after one year than Stage I & II patients (Hammerlid et al, 1997, 1998). However, these studies have also shown that patients who died within the first year after diagnosis, had scored significantly worse than survivors on the majority of domains, already at diagnosis. Thus, it is likely that this finding simply reflects the fact that patients who scored worst at diagnosis, i.e. patients with Stage III+IV and those with progressive disease and relapses, had died during the follow-up period.

All but the physical functioning and general health scales varied between tumour sites, Table 3. The laryngeal cancer patients had a tendency to score slightly better on all four of the SF-36 mental health scales. This may possibly be explained by the fact that most (75%) laryngeal tumours were discovered at an early stage and could therefore be treated with one modality (radiation therapy).

Older patients (>65 years) scored worse than norms on all SF-36 scales, while younger patients had values equal to or better than norms on all but three scales (Figures 2A and B). Funk et al have previously shown that head and neck cancer patients scored worse for the majority of SF-36 domains compared to SF-36 normative data at diagnosis and 6 months and that the difference was larger between the younger patients and reference data than for the older (Funk et al, 1997). The result in this study is, however, in line with a previous longitudinal HRQL study using the EORTC QLQ-C30 and QLQ-H&N35 (Hammerlid, Head and Neck, in press) and confirms results from an earlier study (Terell et al, 1999). In the first study we found that the younger patients (<65 years) improved more during the 3 year long observation period than the older patients, i.e. they seem to have a better rehabilitation potential. The discrepancy between the results of this study and Funk's might therefore be explained by the difference in length of follow-up.

### CONCLUSION

Three years after diagnosis the overall quality of life of the H&N cancer patients, measured by the SF-36, was generally comparable to age- and sex-matched normative values for the Swedish population. However, H&N cancer patients reported significantly more role limitations due their physical health, as well as more disease/treatment-related symptoms and problems.

We therefore conclude that despite enduring and possibly lifestyle-limiting problems/symptoms related to H&N cancer and its treatment, the physical and mental health reported by H&N cancer survivors is otherwise unencumbered 3 years after diagnosis compared to their counterparts in the general population – largely irrespective of tumour site or disease stage.

*© 2001 Cancer Research Campaign*

### ACKNOWLEDGEMENTS

This study was made possible by grants from the Assar Gabrielsson Foundation, King Gustav V, Jubilee Clinic Cancer Research Foundation in Göteborg, the Swedish Cancer Society, the Göteborg Medical Society and the Medical Faculty, Göteborg University. Special thanks to Nils-Gunnar Pehrsson and Henric Ahlbom for statistical advice, to Ewa Silander for collecting data and to Magnus Ruth and Per Svensson for sharing the normative data for the H&N module with us.

### REFERENCES

Aaronson NK, Ahmedzai S (1993) The European Organization for Research and Treatment of Cancer QLQ-30: A Quality-of-Life Instrument for Use in International Clinical Trials in Oncology. *J Natl Cancer Inst* **85**: 365–376

Bjordal K and Kaasa S (1992) Psychometric validation of the EORTC Core Quality of Life Questionnaire, 30-item version and a diagnosis-specific module for head and neck cancer patients. *Acta Oncol* **31**(3): 311–321

Bjordal K and Kaasa S (1995) Psychological distress in head and neck cancer patients 7–11 years after curative treatment. *Br J Cancer* **71**: 592–597

Bjordal K, Ahlner-Elmqvist M (1994a) Development of a European Organization for Research and Treatment of Cancer (EORTC) questionnaire module to be used in quality of life assessments in head and neck cancer. *Acta Oncol* **33**(8): 879–885

Bjordal K, Kaasa S (1994b) Quality of life in patients treated for head and neck cancer: A follow-up study 7 to 11 years after radiotherapy. *Int J Radiation Oncology Biol Phys* **28**(4): 847–856

Bjordal K, Freng A (1995) Patients self-reported and clinical-rated quality of life in head and neck cancer patients: a cross-sectional study. *Oral Oncol Eur J Cancer* **31B**: 235–241

Bjordal K, Hammerlid E (1999) Quality of life in head and neck cancer patients: validation of the EORTC QLQ-H&N35 *J Clin Oncology* **17**: 1008–1019

Bradley JV (1968a) Distribution-Free Statistical Tests. JV Bradley. London, Prentice-Hall: 73–76

Bradley JV (1968b) Distribution-Free Statistical Tests. JV Bradley. London, Prentice-Hall: 78–80

Chin MH and Goldman L (1998) Gender differences in 1-year survival and quality of life among patients with congestive heart failure. *Med Care* **36**: 1033–1046

de Boer MF, McCormick LK (1999) Physical and psychosocial correlates of head and neck cancer: A review of the literature. *Otolaryngol Head Neck Surg* **120**: 427–436

de Graeff A, de Leeuw JRJ (1999a) A prospective study on quality of life of patients with cancer of the oral cavity or oropharynx treated with surgery with or without radiotherapy. *Oral Oncology* **35**: 27–32

de Graeff A, Leeuw RJ (1999b) A prospective study on quality of life of laryngeal cancer patients treated with radiotherapy. *HeadNeck* **21**: 291–296

Deleyiannis FW-B, Wey u, Ernest A (1997) Quality of Life of Disease-free Survivors of Advanced (stage III or IV) Oropharyngeal Cancer. *Head Neck* **19**: 466–473

Deleyiannis FW, Weymuller EA (1999) Quality of Life after laryngectomy: Are functional disabilities important? *Head Neck* **21**: 319–324

Einhorn J, Frödin JE (1996) Strålbehandling vid cancer, volym 1 och 2. Stockholm, SBU Statens beredning för utvärdering av medicinsk metodik (The Swedish Council on Technology Assessment in Health Care).

Fayers P, Aaronson NK, (1995) EORTC QLQ-C30, Scoring Manual, Belgium: EORTC Data Center

Funk G, Hynds L, (1997) Baseline and post-treatment assessment of the general health status of head and neck cancer patients compared with United States population norms. *Head & Neck* **19**: 675–683

Hammerlid E (1997) Quality of Life in Head and Neck Cancer, Longitudinal Evaluation During Treatment and Rehabilitation. *Otorhinolaryngology, Head and Neck Surgery*. Göteborg, University of Göteborg, Sweden: 240

Hammerlid E, Bjordal K (1997a) A prospective longitudinal quality of life study of patients with head and neck cancer. *Otolaryngol Head Neck Surg* **116**(6): 666–673

Hammerlid E, Mercke C (1997b) A Prospective Quality of Life Study of Patients with Oral or Pharyngeal Carcinoma Treated with External Beam Irradiation with or without Brachytherapy. *Eur J Cancer, Oral Oncol* **33**(3): 189–196

Hammerlid E, Mercke C (1998a) A Prospective Quality of Life Study of Patients with Laryngeal Carcinoma by Tumourstage and Different Radiation Therapy Schedules. *The Laryngoscope* **108**(5): 747–759

Hammerlid E, Persson LO (1998b) Quality of Life of Psychosocial Intervention in Head and Neck Cancer Patients. *Otolaryng Head Neck Surg* **120**(4): 507

Hammerlid E, Wirblad B (1998c) Malnutrition and Food Intake in Relation to Quality of Life in Head and Neck Cancer Patients. *Head & Neck* **20**: 540–548

Hammerlid E, Bjordal K (1999) Prospective Multicentre Study in Sweden and Norway of Mental Distress and Psychiatric Morbidity in Head and Neck Cancer. *BJC* **80**(5/6): 766–774

Hjermstad MJ, Fossa S (1995) Test/Retest Study of the European Organization for Research and Treatment of Cancer Core Quality-of-Life Questionnaire. *J Clin Oncol* **13**(5): 1249–1254

Hjermstad M, Fayers P (1998a) Using reference Data on Quality of Life-the Importance of Adjusting for Age and Gender, Exemplified by the EORTC QLQ-C30 (+3). *Eur J Cancer* **34**(9): 1381–1389

Hjermstad MJ, Fayers PM (1998b) Health-Related Quality of Life in the General Norwegian Population Assessed by the European Organization for Research and Treatment of Cancer Core Quality-of-Life Questionnaire: The QLQ=C30 (+3). *J Clin Oncol* **16**: 1188–1196

Jones E, Lund VJ (1992) Quality of life of patients treated surgically for head and neck cancer. *J Laryngol Otol* **106**(March): 238–242

Karnofsky DA, Abelman WH (1948) The use of nitrogen mustards in the palliative treatment of carcinoma. *Cancer* **1**: 634–656

King MT (1996) The interpretation of scores from the EORTC quality of life questionnaire QLQ-30. *Qual Life Res* **5**: 555–567

List MA, Ritter-Sterr CA (1996) Longitudinal Assessment of Quality of Life in Laryngeal Cancer Patients. *Head&Neck* **18**: 1–10

List MA, Siston A (1999) Quality of Life and Performance in Advanced Head and Neck Cancer Patients on Concomitant Chemoradiotherpy: A Prospective Examination. *J Clin Oncol* **17**: 1020–1028

Morton RP (1997) Laryngeal cancer: Quality-of-life and cost-effectiveness. *Head Neck* **19**: 243–250

Osborne ML, Vollmer WM (1998) Characteristics of Patients with Astma Within a large HMO. *Am J Respir Care Med* **157**: 123–128

Osoba D, Zee B (1994) Psychometric properties and responsiveness of the EORTC Quality of Life Questionnaire (QLQ-C30) in patients with breast, ovarian, and lung cancer. *Qual Life Res* **3**: 353–364

Osoba D, Rodrigues G (1998) Interpreting the Significance of Changes in Health-Related Quality of life Scores. *J Clin Oncol* **16**: 139–144

Pruyn JFA, Jong PC (1986) Psychosocial aspects of head and neck cancer-a review of the literature. *Clin Otolaryngeol* **11**: 469–474

Rogers S, Lowe D (1999) The University of Washington Head and Neck Cancer measure as a Predictor of Outcome Following Århmary Surgery for Oral Cancer. *Head Neck* **21**: 394–401

Sullivan M, Karlsson J (1994) SF-36 Hälsoenkät. Svensk Manual och Tolkningsguide. (Swedish Manual and Interpretation Guide). Göteborg, Sahlgrenska University Hospital

Sullivan M, Karlsson J (1995) The Swedish SF-36 Health Survey I. Evaluation of data quality, scaling assumption, reliability and contruct validity across general population in Sweden. *Soc Sci Med* **41**: 1349–1358

Terell J, Nanavati K (1999) Health impact of head and neck cancer. *Otolaryngol Head Neck Surg* **120**: 852–859

Ware JE and Sherbourne DC (1992) The MOS 36-Item Short-Form Health Survey (SF-36). *Med Care* **30**(6): 473–483

Ware JE, Snow KK, (1993) SF-36 Health Survey Manual and Interprentation Guide. Boston, New England Medical Center, The Health Institute

 © 2001 Cancer Research Campaign
